# Habitat use and sex-specific foraging behaviour of Adélie penguins throughout the breeding season in Adélie Land, East Antarctica

**DOI:** 10.1186/s40462-015-0052-7

**Published:** 2015-09-21

**Authors:** Michel Widmann, Akiko Kato, Ben Raymond, Frédéric Angelier, Benjamin Arthur, Olivier Chastel, Marie Pellé, Thierry Raclot, Yan Ropert-Coudert

**Affiliations:** Ecole Normale Supérieure de Lyon, 46 allée d’Italie, 96364, Lyon, Cedex 07 France; CNRS, UMR7178, 67037 Strasbourg, France; Université de Strasbourg, IPHC, 23 rue Becquerel, 67087 Strasbourg, France; Australian Antarctic Division, Department of the Environment, Australian Government, Channel Highway, Kingston, 7050 Australia; Centre d’Etudes Biologiques de Chizé, CNRS UPR 1934, 79360 Villiers-en-Bois, France; Institute for Marine and Antarctic Studies, University of Tasmania, Hobart, TAS 7001 Australia

**Keywords:** Adélie penguin, Marine Protected Areas, Bio-logging, Foraging behaviour, Sea-ice distribution, Polynya, Adélie Land

## Abstract

**Background:**

Marine predators are ecosystem sentinels because their foraging behaviour and reproductive success reflect the variability occurring in the lower trophic levels of the ecosystem. In an era of environmental change, monitoring top predators species can provide valuable insights into the zones of ecological importance that need to be protected. In this context, we monitored the Adélie penguin (*Pygoscelis adeliae*) as a bio-indicator near Dumont d’Urville, an area of the East Antarctic sector currently being considered for the establishment of a Marine Protected Area (MPA), using GPS-based tracking tags during the 2012/13 austral summer breeding season.

**Results:**

The habitat use and foraging areas of the penguins differed by breeding stage and sex and were strongly associated with patterns in bathymetry and sea-ice distribution. The first trips, undertaken during the incubation phase, were longer than those during the guard phase and were associated with the northern limit of the sea-ice extent. During the guard phase, birds strongly depended on access to a polynya, a key feature in Antarctic marine ecosystem, in the vicinity of the colony. The opening of the ice-free area was synchronous with the hatching of chicks. Moreover, a sex-specific use of foraging habitat observed only after hatching suggests sex-specific differences in the diet in response to intra-specific competition.

**Conclusions:**

Sea-ice features that could be affected by the climate change were important factors for the use of foraging habitat by the Adélie penguins. The extent of the foraging area observed in this study is congruent with the area of the proposed MPA. However, both penguin behavior and their environment should be monitored carefully.

## Background

Changes in climatic variables of the atmosphere and ocean system are unequivocal and are occurring at alarming rates. An average 1.5 °C increase in the air temperature is expected worldwide by the end of the 21 century [1]. Since climatic variables drive many ecological networks, biological ecosystems may be affected worldwide through changes in resource availability, phenology, migration and habitat destruction [2]. Marine ecosystems integrate an important range of effects of change in environmental variables, such as temperature, ultraviolet radiation, acidity, and salinity changes, and represent therefore a relevant study field of global shifts. Studies assessing the effects of climate change in these environments are, however, underrepresented compared with those of terrestrial ecosystems [3]. The strongest and fastest signals of global change take place in polar regions, where temperatures are rising [4] and loss of mass of the ice sheets and glacial retreat are accelerating [5]. These environments are thus relevant study areas of global changes. Several international organizations, such as the Scientific Committee for Antarctic Research (SCAR) and the Commission for the Conservation of Antarctic Marine Living Resources, have actively focused on conservation programs of these polar environments by providing scientific information that could help establish Marine Protected Areas (MPAs) in East Antarctica [6]. During the 1^st^ SCAR Horizon Scan conducted in April 2014, two of the 80 questions selected to be representative of the scientific domains that need to be explored in the coming 50 years were specifically focused on the need to examine the relevance of MPAs to conservation of the marine resources of the Southern Ocean [7]. Therefore, relevant eco-regionalization information [8] is needed for reappraisal of these protected areas (and elsewhere too) since the situation is constantly evolving.

Yet monitoring a whole ecosystem is logistically difficult, if not impossible, in these remote regions of the globe. To address this issue, ecologists use sentinel species, especially meso- and top predators like seabirds and marine mammals, as they integrate and amplify effects occurring at lower levels of the food web [9–11]. The use of tracking devices [12] allows scientists to study behaviors occurring in distant areas and represents a powerful approach for conservationists [13]. Animal geographic positions through space and time offer insights into where individuals travel, forage, rest and interact with each other, and thus give a better understanding of the functioning of marine ecosystems. Moreover, these data can be spatially and temporally linked with physical parameters in order to examine the animals’ responses to environmental constraints [11]. Consequently, areas of importance to meso- and top predators are an important consideration for the establishment and evaluation of MPAs [14, 15].

Adélie penguins (*Pygoscelis adeliae*) have been well studied in Antarctica over the past four decades. Their main prey are Antarctic krill (*Euphausia superba*), ice krill (*E. crystallorophias*) and Antarctic silverfish (*Pleuragramma antarcticum*), whose abundance is strongly dependent on primary production and sea-ice concentration, extent, and distribution [16, 17]. Adélie penguins breed during the short austral summer and are strongly impacted by changing sea-ice conditions, e.g. [18, 19], and thus can be considered as a relevant bio-indicator of global environmental changes and Antarctic marine ecosystem health [20]. The spatial distribution of Adélie penguins at sea has been studied around the continent in several instances, e.g. [21–23], and has helped to understand the response of the Antarctic ecosystem to change. Here, we investigate the spatial distribution and foraging patterns of Adélie penguins throughout their breeding season at the colony of Ile des Pétrels (Dumont d’Urville station) in Adélie Land, East Antarctica. Preliminary studies in this region have highlighted the general trends in the spatial at-sea distribution of Adélie penguins, but tracked only a small number of birds using relatively imprecise satellite trackers [16], or were conducted with more precise positioning loggers (GPS) but on males only and only during the incubation phase [24]. The use of satellite trackers that deliver positions of relatively low precision can potentially lead to large errors in home range definition [25]. Here, using miniature GPS loggers, we examined the at-sea distribution and foraging activity of both male and female Adélie penguins during different stages of their breeding period when feeding requirements differ, i.e. during incubation, early and late guard stages. The aim of the study was also to investigate the influence of environmental factors, such as water depth and of changing sea-ice conditions, and sex on the at-sea behaviour and habitat use of Adélie penguins throughout the season, so as to refine the scientific information that can serve as an evaluation of the proposed MPA off Adélie Land, i.e. the D’Urville Sea-Mertz MPA [6].

## Methods

### Field procedure

The study was conducted near Dumont d’Urville station, Adélie Land (66°40’S, 140°01’E), Antarctica, between November 18, 2012 and January 17, 2013. At the beginning of the breeding season, around October, males travel southwards through the pack ice to reach their colony. After the courtship, two eggs are laid. The incubation starts once the second egg is laid and lasts 32 – 34 days. Males undertake the first shift while their mate forages at sea for 11 to 14 days. After the eggs hatch, chicks are cared for by both parents for ca. 22 days during what is referred hereafter as the guard phase. Parents take turns between foraging at sea and guarding their offspring. We separated the guard phase into the early (mid-December to early January) and the late guard phases (from early January to mid-January) to account for the growing demand from the chicks and its possible influence on trip duration. When the chicks become thermally independent around mid January, they gather in crèches. At this stage, parents forage simultaneously and feed their chicks every 1 to 2 days until they fledge at the age of 50–60 days in February [26].

At the courtship, birds were marked on the breast with Nyanzol dye for identification in the colony and for the monitoring of their breeding activity throughout the season. At this occasion, observations were made and birds were sexed based on behaviour. A total of 65 birds were captured and equipped with CatLog™ GPS loggers (16 Mb, memory, 380 mA lithium-ion battery, Catnip Technologies, USA) customized at our laboratory by the engineers of MIBE (IPHC-CNRS, UMR7178, Strasbourg, France) as described in [24]. The loggers were placed in waterproof heat-shrink tubes (final weight: 30 g, final size: 14 × 35 × 70 mm, covering 1.7 % of the bird’s cross-sectional area). The devices were set on the birds’ lower back feathers using mastic and waterproof adhesive Tesa® tape, and tightened up with two Colson® plastic clamps. Since the battery life and memory capacities were limited, loggers were programmed to record time, latitude and longitude every 30 min during incubation and every 3 min during the guard stage. Studied birds were tracked only for a single trip to minimize any potential disturbance caused by the device or handling. The nests of equipped animals were kept under surveillance every 1–4 h until the return of the bird. Individuals were then recaptured outside of or on their nest, loggers removed and data downloaded using @trip PC software (http://global.mobileaction.com/download/i-gotU_download1.jsp).

Of the 65 loggers, 22 were deployed during incubation (11 females and 11 males), 23 during the early guard phase (10 females and 13 males), and 20 during the late guard phase (10 females and 10 males) (Table 1). Although 12 loggers malfunctioned and recorded incomplete tracks, based on the time spent at sea we estimated that at least up to 70 % of the trip had been recorded in six of these twelve cases, the last 30 % corresponding to the return phase of the trip. Thus, we chose to keep these six birds in the analyses given that removing them did not modify our conclusions.

### Data analysis

GPS coordinates were first processed using IGOR Pro 6.12A (Wavemetrics, USA). Duplicated coordinates, excess points before departure and after arrival at the colony were removed. Then, spatial analyses were conducted using R 3.0.1 software (R Development Core Team; www.R-project.org), using the adehabitat LT [27] and sp packages. The total distance traveled was calculated by adding the distances between consecutive GPS coordinates over the entire track of an individual trip.

For first-passage time analyses, positions were interpolated to a regular step length of 500 m for incubation stage trips, and 50 m for guard stage trips. First-passage time (FPT) is defined as the time an animal requires to cross a circle of a given radius [28]. This method detects speed and tortuosity changes in movement patterns along a trajectory and therefore indicates areas of concentrated foraging activity known as area-restricted search (ARS) behaviour. These ARS areas are assumed to be related to aggregations in the spatial distribution of prey [28]. Several radius sizes were tested in order to find an appropriate spatial scale for ARS [29]. Radius values from 50 to 20 000 m were tested for incubation stage trips, and from 5 to 1000 m for guard stage trips. FPT was then calculated separately for each bird at each trip location. The appropriate circle size (i.e. the spatial scale of the intensively searched area) was selected by finding the radius associated with the peak in the plot of the variance of log-transformed FPT against radius S(r). If several peaks were detected, the smallest radius was chosen, because large values may tend to classify the whole open-water section of the trip as ARS [29].

### Environmental features

We used bathymetry data (ocean depth at one-minute horizontal spatial resolution; [30]) and passive-microwave estimates of daily sea-ice concentration (12.5 × 12.5 km resolution) from the Institut Français de Recherche pour l'Exploitation de la Mer (Ifremer, ftp://ftp.ifremer.fr/ifremer/cersat/products/gridded/psi-concentration/data/antarctic/daily/netcdf/). The sea ice data do not allow fast and pack ice to be reliably distinguished, and so we did not attempt to do so here. Data collected between 63-67°S and 134-143°E were analyzed and converted to maps using the raster and fields packages in R. Depth and daily sea ice concentration values were extracted for each location on each track using bilinear interpolation from the native ice and depth grids.

### Statistics

Statistical tests were conducted using R. The effects of gender and breeding stage on trip length (time spent at sea) and total distance travelled were tested using non-parametric Kruskal-Wallis tests, followed by Mann–Whitney rank sum tests. Statistical significance was assumed under a p-value threshold < 0.05. Normality of time spent at sea and total distance traveled was assessed with the Shapiro-Wilk test. Trips during incubation and guard stage were significantly different and, hence, were analyzed separately. Sea-ice concentration values were compared between genders and stages using Gaussian linear mixed models with random intercept by individual bird to account for repeated measurements of the same individual.

## Results

### Area coverage

**Fig. 1:**
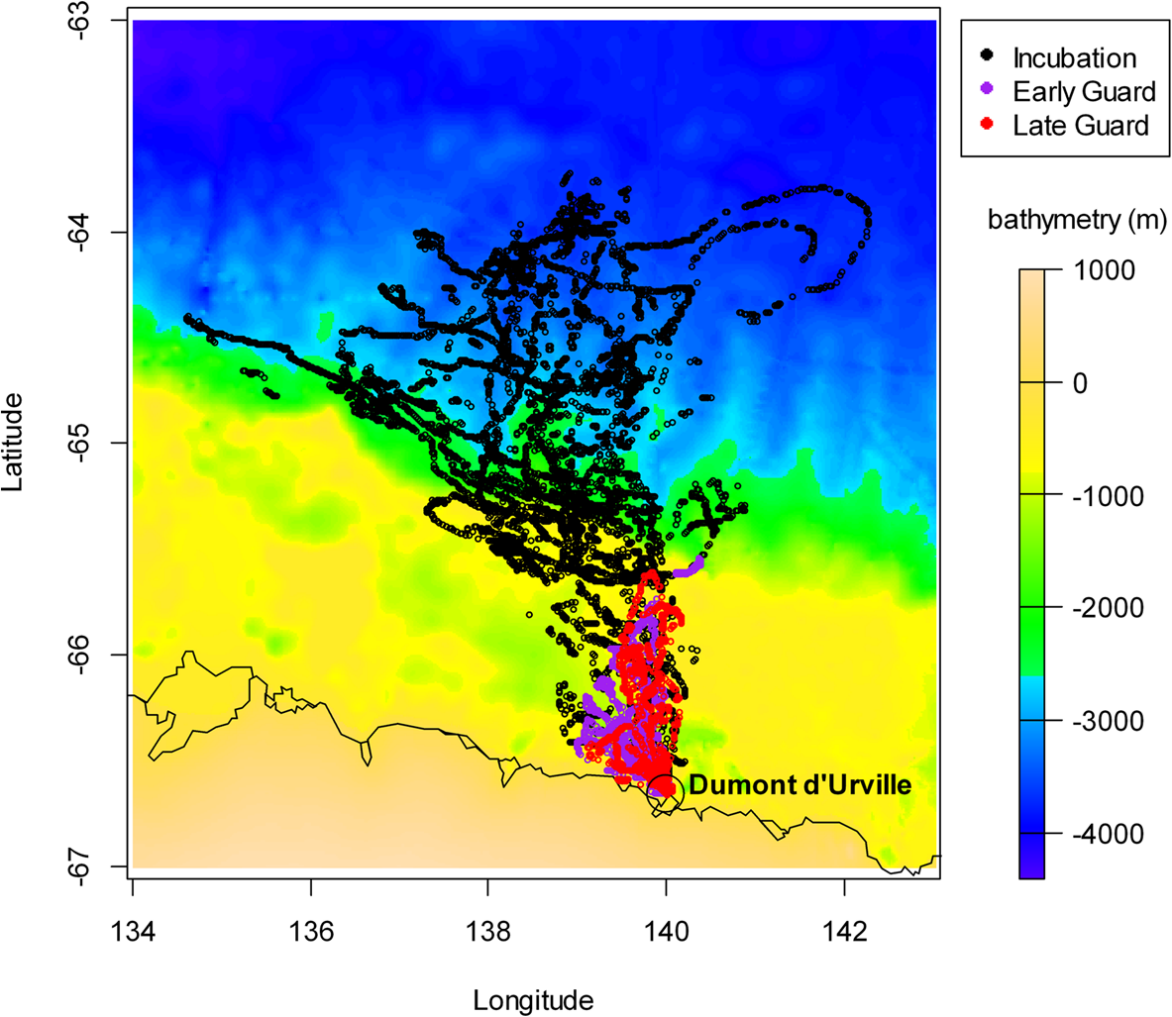
Bathymetry (from Smith and Sandwell 1997) of Dumont d’Urville area overlayed with penguin foraging trips. Colours indicate incubation (black), early guard (red) and late guard (purple) phases. Each point corresponds to one logger record; colours indicate breeding stages, identity of birds isnot colour-discriminated. Depth signal corresponds to land (1000 to 0m), continental shelf (0 to -900m),continental slope (-1000 to-2500m) and the abyssal plain (below -2500m)

The spatial coverage of the foraging trips ranged from 63.7°S to 66.6°S, and from 134.7°E to 142.3°E, corresponding to an area of 119 389 km^2^ (Fig. 1). Temporal coverage extended over a 60-day period from November 18, 2012 to January 17, 2013. Penguins undertook longer trips during incubation than during guard (mean maximum distances 287 km ± 54 km and 57.6 km ± 45 km, respectively; *W* = 3, *p* < 0.001). During incubation, the birds reached the edge of the continental shelf, the slope and eventually the open ocean of the Dumont d’Urville Sea, whereas foraging trips were confined to the shallower neritic waters of the continental shelf during the early and late guard phase. Some penguins followed the continental shelf break around 64.8°S. This trend was particularly noticeable among birds heading north-west. One penguin foraged farthermost westward along the shelf break (to ~134.5°E, Fig. 1), and the logger battery expired before the end of the trip.

**Fig. 2:**
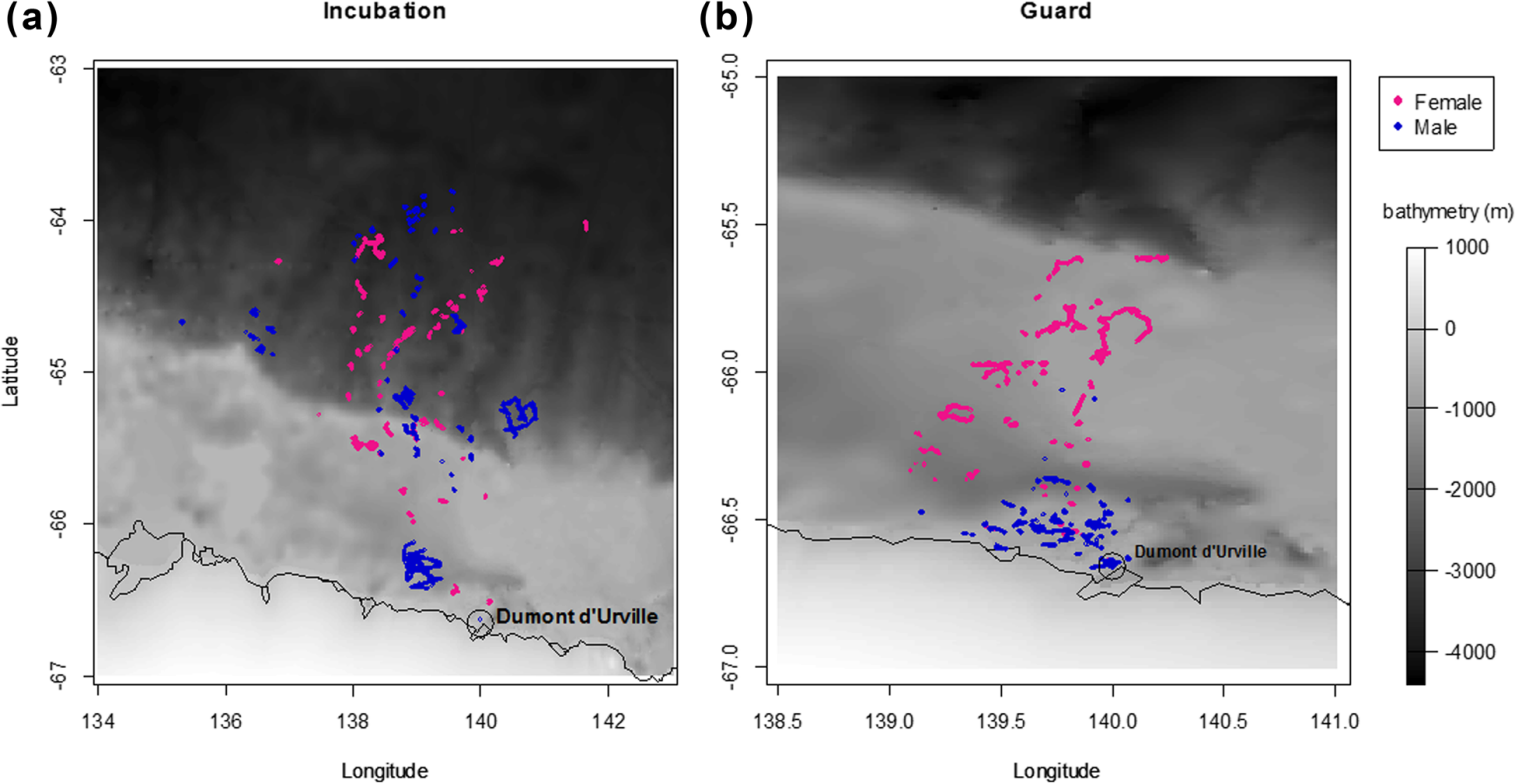
Location of ARS of female and male Adélie penguin during (**a**) incubation and (**b**) guard phase.Note that the scale is magnified for the guard phase when birds foraged in the vicinity of the colony

ARS were detected in small patches spread along all trips and did not necessarily occur in the most remote areas that penguins reached (Fig. 2a, b). The penguins regularly alternated between transit and foraging behavior during the course of a trip. ARS positions indicate that females foraged in more remote locations than males during the guard phase (Fig. 2b).

### Sea-ice distribution and spatial adjustment in habitat use by birds

**Fig. 3:**
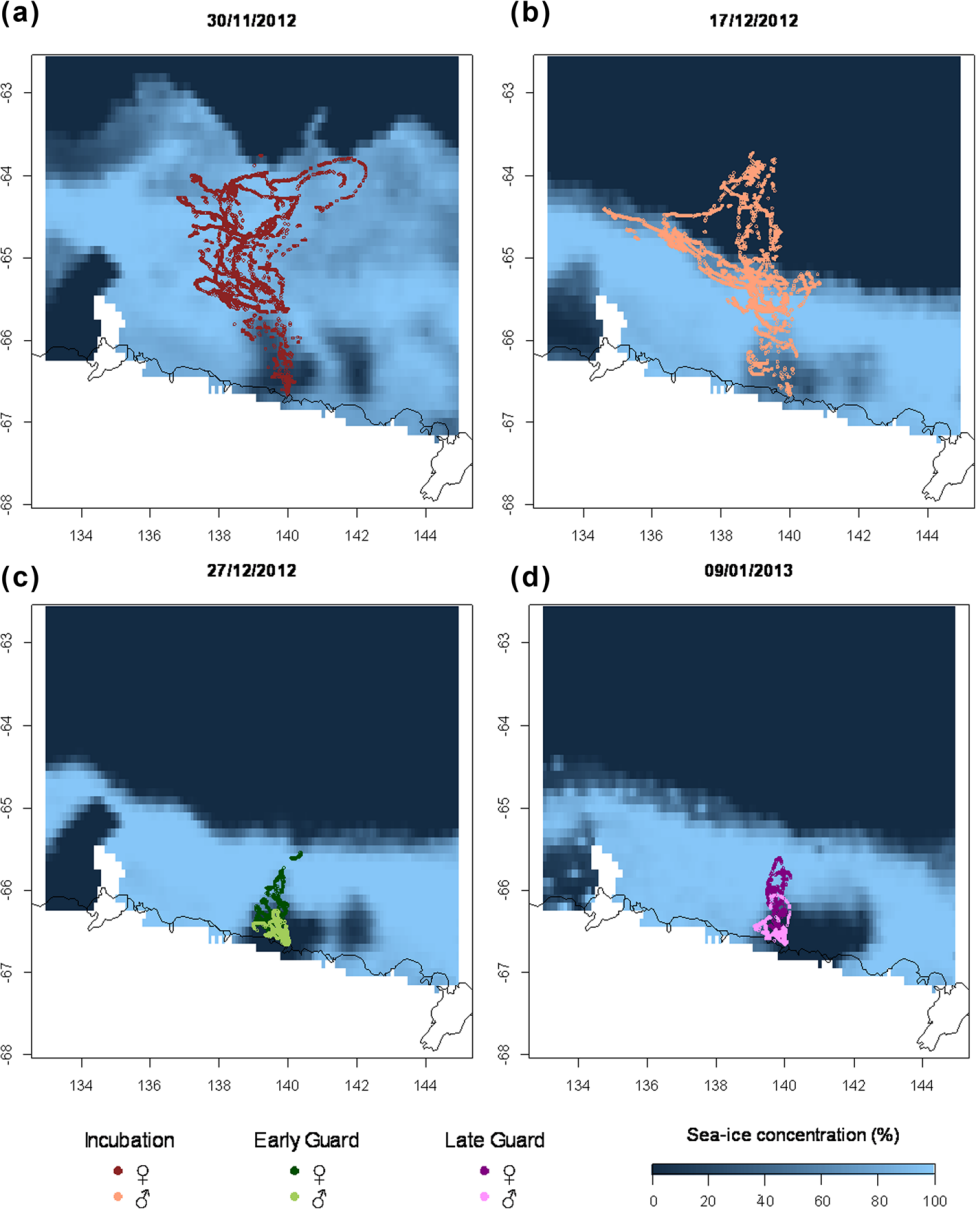
Location of foraging birds in Dumont d’Urville area with sea-ice coverage at four different dates.Ice scale corresponds to concentration of fast ice percentage, 0 % being open water and 100 % full ice coverage. Colours indicate breeding stages and genders (female incubation trips (**a**), male incubation trips (**b**), early guard (**c**) and late guard (**d**))ᅟ

At the beginning of the breeding season in mid-November, ice coverage extended to 63.5°S so that females had to perform longer trips to reach the sea-ice edge and open water (Fig. 3a). At the beginning of December, the sea ice cleared in the north-east of the region so that an ice-free area appeared at 64.5°S, east of about 138°E. When the males started their first foraging trips of the incubation phase the northernmost edge of the sea ice was at approximately 65°S, roughly 100 km closer to the colony than it was in mid-November. On their return journey, all males followed the sea-ice edge before heading to the colony on a straight course. Only one bird did not forage in the ice-free area but followed this border during its entire trip. Penguins guarding chicks foraged in an area of open water completely surrounded by sea ice (polynya) that had opened by the end of November in the vicinity of the colony, between 65.6°S and the continent. This polynya offered an area of approximately 10,000 km^2^ to forage, corresponding to 8.4 % of the total area explored by penguins during incubation.

### Differences between stages and between genders

**Fig. 4:**
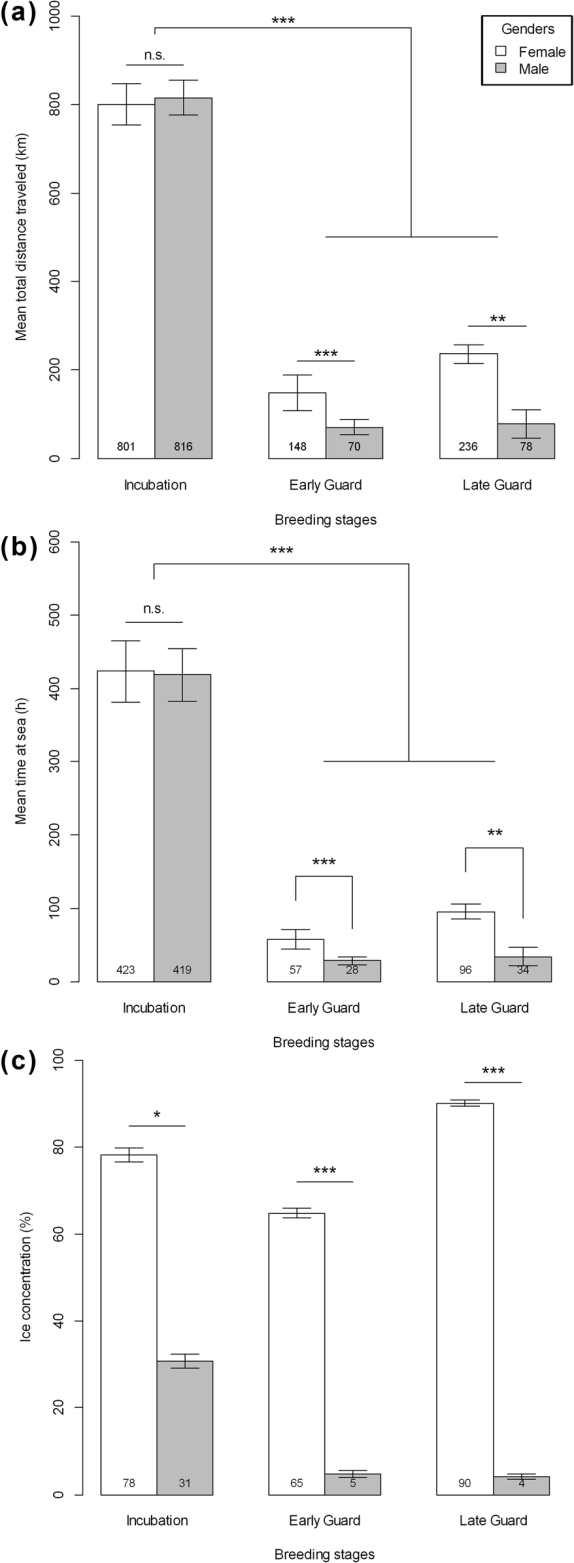
(**a**) Mean total distance travelled, (**b**) mean time spent at sea and (**c**) sea-ice concentration in ARS according to breeding stages and genders. Standard deviation bars and mean values are displayed. Significance is assumed for p-values as (*) from 0.05 to 0.01, (**) from 0.01 to 0.001 and (***) below 0.001

**Fig. 5:**
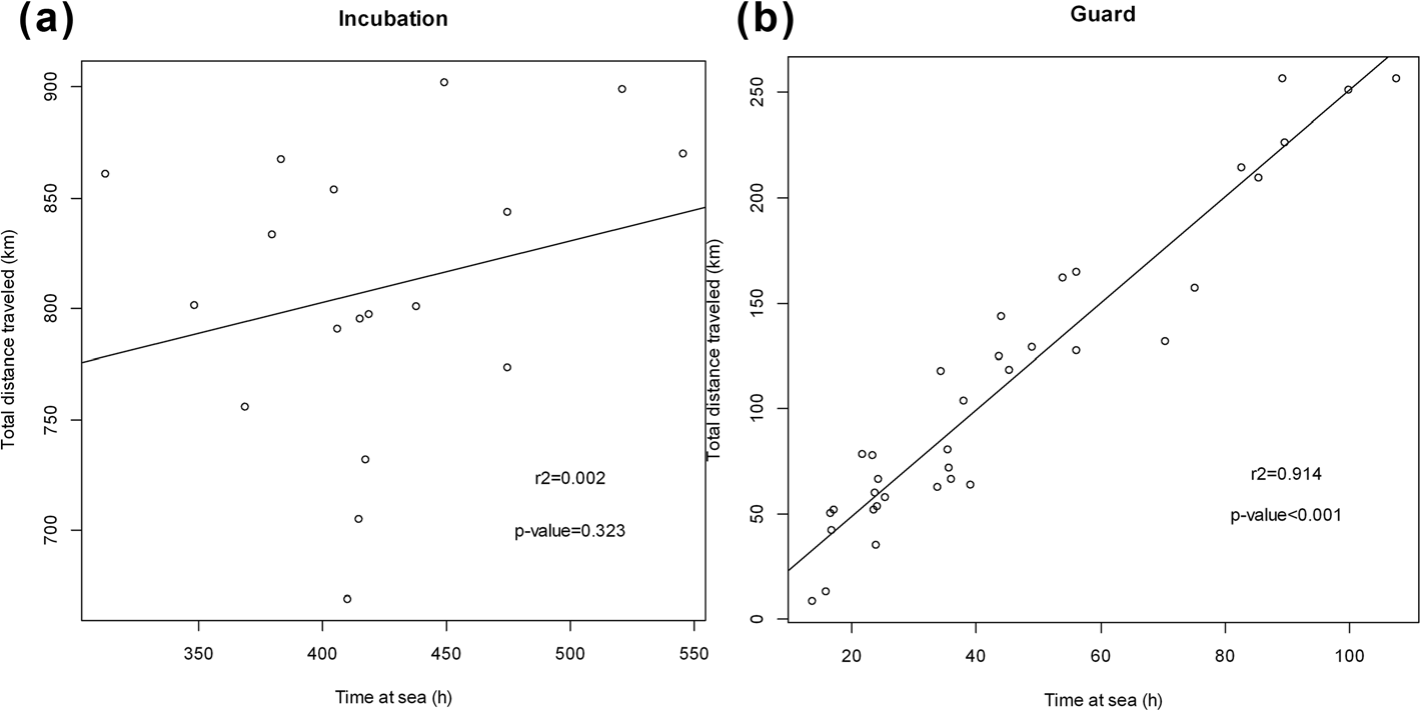
Relation between total distance travelled and time spent at sea during (**a**) incubation and (**b**) guardphases

The mean time spent at sea was significantly different between stages (Fig. 4b, *χ*^2^ = 43.0, df = 5, *p* < 0.001). Trips were longer during incubation than during both early and late guard stages (W = 396, *p* <0.001 and W = 234, *p* < 0.001, respectively) but did not differ between the two guard stages (W = 165, *p* = 0.468). During incubation, males and females foraged for a similar duration (W = 47, *p* = 0.605), but during the two guard phases females spent more time at sea than males (W = 106, *p* < 0.001 and W = 36, *p* = 0.002, respectively). Similarly, the mean total distance traveled by the birds also varied throughout the season (Fig. 4a, *χ*^2^ = 42.8, df = 5, *p* < 0.001). The distances were longer during incubation than during both early and late guard (W = 396, *p* < 0.001 and W = 234, *p* < 0.001, respectively), but the difference was not significant between early and late guard phases (W = 155, *p* = 0.7). There was no difference in distances travelled during incubation (W = 39, *p* = 0.931) but females travelled longer distances during the early (W = 106, *p* < 0.001) and late guard (W = 36, *p* = 0.003) stages. Time spent at sea and total distance traveled were positively correlated during guard stages (Pearson r^2^ = 0.914, *p* < 0.001, Fig. 5b) but, unexpectedly, no relationship was found between these two variables during incubation (r^2^ = 0.002, *p* = 0.323, Fig. 5a). Foraging trips during guard stage were more direct and less tortuous than the long incubation trips.

Females encountered significantly higher sea-ice concentration in the foraging areas than males (Fig. 4c) during incubation (*p* = 0.044), and during early and late guard phases as well (*p* < 0.001 for both). During incubation, females and males encountered different sea-ice conditions because they foraged at different periods, females foraging in areas with extensive sea ice which had recessed when males foraged. During guard stage, males and females foraged at the same time, i.e. ice distribution in the environment was similar for females and males and, as such, greater ice concentrations along the route of the females suggest a sex-specific selection of foraging sites (Fig. 3c, d). The differences between the sexes were greater than between the stages.

## Discussion

This study showed that the habitat use and foraging strategies of Adélie penguins changed throughout the breeding season according to a key environmental driver: sea-ice distribution. The sea-ice edge is indeed a critical foraging habitat for Adélie penguins during the incubation phase in this sector of the Antarctic. During incubation, birds relied on a distant sea-ice edge. Then, the extent of the foraging area decreased more than 10 times during the guard phase when the birds restricted their foraging to the polynya area adjacent to the colony. A second driver of foraging behavior and breeding investment for all breeding stages was sex, with females spending more time at sea and traveling longer distances after the eggs hatched. Additionally, during the guard phase females spent more time at sea and traveled longer distances than males. This sex-discrepancy may suggest a difference in breeding investment. Our results are consistent with the observations made on the foraging activity of Adélie penguins from Béchervaise Island [22], which showed similar patterns in foraging range and strategy.

### Environmental conditions and breeding stages

As sea-ice distribution changed throughout the succession of shifts taken by males and females, the penguins used different habitats. Females reached more northern areas than males in mid-November, at the beginning of the incubation phase, when the sea ice was most extensive. When males started travelling in early December, the sea-ice edge had retreated and the males showed more flexibility in their at-sea exploration. Incubation foraging trips were shorter during seasons when the sea ice retreated earlier [17]. Furthermore, densities of Antarctic krill (a principal food source of Adélie penguins, e.g. [16]) tend to be elevated just south of the northern sea-ice edge [31]. As the sea ice melts and retreats, the ice cover over the shelf break and slope is reduced and these areas become available for foraging. This typically occurs sometime during the month of December in this region. The topography of the shelf break causes deep currents to be deflected towards the surface, creating an upwelling that further enhances nutrients and therefore productivity in the near-surface layer. Because this zone is characterized by these two oceanographic features (the upwelling from the shelf break and the melting from the sea-ice edge), it provides a profitable foraging ground for two main reasons. First, krill is highly concentrated, reducing travel and search time and consequently lowering energy expenditure compared with areas of dispersed prey. Secondly, although its distance from the colony may vary, the sea-ice edge represents an area of predictability where prey can reliably be expected to be available across seasons [32]. Oceanic currents do not lead to nutrient redistribution only. While currents and winds drive sea-ice dispersal and thus impact indirectly the birds’ foraging movements, birds have also been observed to use offshore-flowing currents to assist the outward leg of foraging trips, and potentially to locate open-water foraging spots around eddies [24].

Later in the reproductive season when parents reared chicks, a coastal polynya opened in the vicinity of the colony. This polynya is a recurring feature, and results from katabatic and synoptic winds from the land, which blow the sea ice in a divergent and persistent direction, leading the sea ice away from the continent [33]. The reduced cover within the sea-ice zone allows the light to penetrate the water and, as a driver of photosynthesis, leads to a proliferation of phytoplankton [34]. Adélie penguin colonies (but also emperor penguin (*Aptenodyptes forsteri*) colonies, [32]) occur in the vicinity of polynyas that provide access to foraging grounds during spring [22, 34–36]. At Dumont d’Urville, incubating Adélie penguins took on average 24 h to reach the continental shelf and the open water. The presence of the coastal polynya allowed the birds to shorten their trips after the eggs hatched, at a time when it is crucial for them to minimize the time spent at sea while maximizing the food provisioning for their chicks [37]. If a breeding parent spends too much time at sea, young chicks, which need to be kept warm and protected against predators, can be abandoned by its partner fasting on land [38]. This further highlights the importance of synchronicity between breeding events (i.e. the hatching and the change in food requirement) and sea-ice retreat (see [17]). The dependence of the Adélie penguins at Dumont d’Urville on this polynya has been dramatically exemplified by the total breeding failure in the 2013/14 season when extensive sea ice and the absence of strong katabatic winds prevented the opening of the polynya [19]. The duration of guard phase trips to the polynya correlated with total distance traveled but this was not true during incubation. This may have been due in part to the open water of the polynya allowing unhindered swimming travel. In contrast, earlier in the season the birds must navigate a heterogeneous sea-ice landscape. The lower tortuosity of tracks during the guard phase compared with the incubation phase further suggests that birds guarding chicks invested their foraging time into traveling without devoting time to exploration. In contrast, during incubation birds did not use their full time at sea to reach the greatest distance possible but spent some periods of time exploring small areas.

When chick-rearing Adélie penguins are foraging in the polynya we expect the intraspecific and interspecific competition to increase to some extent. In some extreme cases, this may constrained the foraging activity of the birds and lead them to search for other – potentially more distant – foraging areas. For instance, Adélie penguins from a Ross Sea colony compete with Weddell seals (*Leptonychotes weddellii*), emperor penguins and Antarctic minke whales (*Balaenoptera bonaerensis*) for the same resource [39]. As a common way of reducing the impact of intraspecific competition, it is not surprising that sex-specific differences in habitat use were observed in our study. Future environmental changes affecting the sea-ice distribution are likely to cause a shift in foraging strategies. For instance, if the polynya opened earlier in the breeding season, resources can be consumed earlier, potentially depleting prey patches earlier and increasing the competitive pressure in the polynya.

### Influence of sex on foraging behaviour

After hatching, females spent longer periods at sea and traveled longer distances than males. In addition, females foraged in areas of higher sea-ice concentration than males independent of the breeding stage. This sex-specific difference in habitat use is probably linked with sex-specific differences in diet. At Béchervaise Island, females feed mainly on Antarctic krill and provide more food to their chicks than males, which feed on fish, mainly Antarctic silverfish [40]. In our study, females were more likely to concentrate their foraging activity close to sea-ice edge where krill should be abundant [31]. Such resource segregation would be an efficient way of reducing the intra-specific competition that is enhanced by the use of the comparatively smaller area of the polynya later in the season.

## Conclusion

Our study shows that the foraging behaviour of Adélie penguins at Dumont d’Urville depends greatly on access to sea-ice edge, and is subsequently enhanced by the presence of a polynya in the vicinity of the colony. Both oceanic features offer concentrated and highly available sources of food. Monitoring this species over the long term would allow us to understand their capacity to adapt to environmental changes that are going to affect these two oceanic features in the future. In addition, the important intra-individual variability observed in the penguins might be driven by parameters other than environmental features and sex alone. Age, experience and physiological conditions might be related to foraging abilities and strategies, e.g. [41, 42], and must be taken into account when assessing the species’ plasticity to change.

Environmental change is not the only threat to Antarctic ecosystems; human impacts, such as commercial fisheries or tourism, are also likely to have an impact [43, 44]. Our data can serve the evaluation of the proposed D’Urville Sea - Mertz MPA from an Adélie penguin perspective. The proposed MPA would become one of four areas of the East Antarctic network of MPAs, extending south of 63.5°S and from 136 to 148°E. Based on our data, this MPA is likely to protect most of the foraging areas of Adélie penguins: during this season, only one bird went just outside the proposed boundaries of the MPA (to 134.7°E). However, during the 2013/14 season, Adélie penguins from Dumont d’Urville had to travel twice the distance to the sea-ice edge than recorded in this study [19]. Such long foraging trips can have a negative impact on chick survival, by forcing them to fast for long periods of time. For this reason, a northward extension of the MPA would be unlikely to be beneficial for the penguins. However, during years with average sea-ice conditions, the proposed MPA would greatly enhance the penguins’ chances to reproduce successfully. Note also our study only covers the reproductive season, and that the area used by penguins during the austral winter is likely to be larger. The non-breeding winter period should not be ignored as Adélie penguins can cross impressive distances between these breeding and non-breeding grounds [23], and further studies on penguins and other seabirds over the winter period are required. In summary, our approach in the determination of foraging habitat of Adélie penguins takes into account the sex and phenology of the species, as well as the environmental factors. Our results support the establishment of the proposed D’Urville Sea-Mertz MPA. As there are obvious biotic and abiotic variations between years, this assessment should be regularly reappraised as part of the MPA monitoring activities.
